# Credibility of Scientists: Industry versus Public Interest

**DOI:** 10.1289/ehp.114-a147a

**Published:** 2006-03

**Authors:** Merrill Goozner

**Affiliations:** Integrity in Science Project, Center for Science in the Public Interest, Washington, DC, E-mail: mgoozner@cspinet.org

In their article “Assessing the Reliability and Credibility of Industry Science and Scientists,” Barrow and Conrad (2006) demonstrated a sophisticated understanding of the nuances of the Federal Advisory Committee Act (1972). They accurately pointed out that the act draws a distinction between conflicts of interest, which hinge on financial self-interest, and bias, which may exist for a host of reasons including research funding sources.

Alas, in their haste to condemn public interest groups who wish the government would adhere to the letter and spirit of that law, Barrow and Conrad (2006) incorrectly characterized objections by the Center for Science in the Public Interest (CSPI) and the Environmenal Working Group (EWG) to two scientists nominated in December 2004 to sit on a U.S. Environmental Protection Agency (EPA) advisory panel evaluating the risk of perfluorooctanoic acid (PFOA) (EWG and CSPI 2004). This misrepresentation may have helped prove their thesis, but it in no way reflects what is actually going on at the U.S. EPA, the National Academies, and other agencies that routinely form advisory panels.

Barrow and Conrad (2006) suggested that the CSPI and the EWG challenged two scientists because they were “funded by industry.” In fact, there were nine industry-funded scientists listed as potential candidates for this panel. The two scientists singled out by the CSPI and the EWG currently or previously worked for DuPont or 3M, which have a direct financial stake in the outcome of the committee’s deliberations (EWG and CSPI 2004). Thus, these scientists were covered by the conflict of interest standard, not the bias standard.

The Federal Advisory Committee Act (1972) states that scientists with conflicts of interest cannot serve on federal advisory committees unless their expertise cannot be recruited elsewhere. The EWG and CSPI (2004) suggested that there were other scientists available with the requisite expertise. The U.S. EPA must have agreed with this analysis, because the final panel announced in February 2005 (U.S. EPA 2005) did not include either scientist, although it did include two others with prior industry ties to whom the groups did not object. By contrast, only one scientist on the panel can be said to be “environmental” in orientation.

Barrow and Conrad (2006) saw this panel as proof that public interest and environmental groups are seeking to tilt the playing field against industry. In fact, industry-funded scientists often play a dominant role on committees established under the Federal Advisory Committee Act (1972). And, as in the PFOA panel case, those with financial support from industry usually outnumber by a two- or three-to-one margin those whose writings suggest they may be sympathetic to environmental or consumer interests (CSPI, in press).

Barrow and Conrad (2006) concluded that industry scientists should be allowed to serve on advisory panels because “they can provide unique knowledge and insight concerning the chemical in question.” No doubt such scientists should be encouraged to present their data to a panel evaluating the health risks of a particular chemical. However, if they work full- or part-time for a company that makes, uses, or competes against the chemical, then allowing those scientists to sit on the panel would be the equivalent of allowing one side in a court case to name the jurors.
